# Modulation of Endocannabinoid Tone in Osteoblastic Differentiation of MC3T3-E1 Cells and in Mouse Bone Tissue over Time

**DOI:** 10.3390/cells10051199

**Published:** 2021-05-14

**Authors:** Magdalena Kostrzewa, Ali Mokhtar Mahmoud, Roberta Verde, Federica Scotto di Carlo, Fernando Gianfrancesco, Fabiana Piscitelli, Alessia Ligresti

**Affiliations:** 1 Endocannabinoid Research Group, Institute of Biomolecular Chemistry (ICB), National Research Council of Italy, 80078 Pozzuoli, Italy; m.kostrzewa@icb.cnr.it (M.K.); amokhtar@icb.cnr.it (A.M.M.); roberta.verde@icb.cnr.it (R.V.); fpiscitelli@icb.cnr.it (F.P.); 2 Institute of Genetics and Biophysics Adriano Buzzati-Traverso, National Research Council of Italy, 80131 Naples, Italy; federica.scotto@igb.cnr.it (F.S.d.C.); Fernando.gianfrancesco@igb.cnr.it (F.G.)

**Keywords:** bone, osteoblasts, endocannabinoids, *N*-acylethanolamines, MC3T3-E1

## Abstract

Bone is a highly complex and metabolically active tissue undergoing a continuous remodeling process, which endures throughout life. A complex cell-signaling system that plays role in regulating different physiological processes, including bone remodeling, is the endocannabinoid system (ECS). Bone mass expresses CB1 and CB2 cannabinoid receptors and enzymatic machinery responsible for the metabolism of their endogenous ligands, endocannabinoids (AEA and 2-AG). Exogenous AEA is reported to increase the early phase of human osteoblast differentiation in vitro. However, regarding this cell context little is known about how endocannabinoids and endocannabinoid-related *N*-acylethanolamines like PEA and OEA are modulated, in vitro, during cell differentiation and, in vivo, over time up to adulthood. Here we characterized the endocannabinoid tone during the different phases of the osteoblast differentiation process in MC3T3-E1 cells, and we measured endocannabinoid levels in mouse femurs at life cycle stages characterized by highly active bone growth (i.e., of juvenile, young adult, and mature adult bone). Endocannabinoid tone was significantly altered during osteoblast differentiation, with substantial OEA increment, decline in 2-AG and AEA, and consistent modulation of their metabolic enzymes in maturing and mineralized MC3T3-E1 cells. Similarly, in femurs, we found substantial, age-related, decline in 2-AG, OEA, and PEA. These findings can expand existing knowledge underlying physiological bone cell function and contribute to therapeutic strategies for preventing bone-related metabolic changes accruing through lifespan.

## 1. Introduction

Bone is a highly intricate and metabolically active tissue that serves indispensable functions such as the structural and mechanical integrity essential for locomotion and protection of vital organs, maintenance of mineral homeostasis, and hematopoiesis. The consonance between bone structure and functionality is maintained by a tightly coordinated remodeling process in which mineralized matrix is removed by osteoclasts and afterward replaced with newly formed bone tissue, produced by osteoblasts [1]. Alteration of such a finely regulated metabolism changes strength or mass of bone structure, leading to bone diseases, such as osteoarthritis or osteoporosis, and to higher fracture risk [2,3,4]. In a murine model of accelerated aging, decreased osteoblastogenesis within bone marrow was associated with diminished bone formation in the remodeling cancellous bone and low bone mineral density (BMD) [5]. The number of osteoblasts in distal femoral metaphysis of male and female C57Bl/6J mice robustly declined from the first and third months of age and remained constant thereafter, while osteoclasts increased in 5-month-old mice before declining at month 12 [6]. However, in vivo bone-forming activity of osteoblasts depends not only on their number but also on their functional lifespan and capability for proliferation and differentiation [2,7]. Indeed, mesenchymal stem cells (MSCs) in bone marrow, which play an essential role in bone physiology giving the origin to osteoblasts alongside chondrocytes or adipocytes, are reported to diminish with age [8]. Age-related loss of cancellous bone and thinning of cortical bone in mice are in line with those occurring in humans [9]. Skeletal structure in mice undergoes substantial changes with advancing age as cancellous bone volume continuously decreases from 1.5 to 24 months of age in C57BL/6J male mice [6,10]. These changes are in line with those occurring in human aging [9]; thus, rodent models serve as suitable tools for studying age-related bone loss.

The endocannabinoid system (ECS) is a complex cell-signaling system that plays a role in regulating different physiological processes, including bone remodeling [11]. Apart from cannabinoid receptors (CB1 and CB2), the ECS includes also two major endogenous ligands (endocannabinoids (ECs)) synthesized “on demand” from lipid precursors known as *N*-arachidonoylethanolamine (anandamide (AEA)) and 2-arachidonoyl glycerol (2-AG) as well as their synthetic and metabolic enzymes [12,13]. Later on, it was realized that other congeners, i.e., *N*-acylethanolamines (NAEs) including *N*-palmitoylethanolamine (PEA) and *N*-oleoylethanolamine (OEA), and 2-mono-acyl-glycerol (2-MAG) and long-chain fatty acid derivatives, counting primary fatty acid amides and several *N*-acylated amino acids, belong to a more complex ECS system called “endocannabinoidome” by sharing redundant biosynthetic pathways and enzymes being involved in a wide range of biological effects [14,15]. The main AEA synthetic pathway comprises phospholipid precursor *N*-arachidonoylphosphatidylethanolamine (NAPE) and its calcium-dependent hydrolysis through an N-arachidonoylphosphatidylethanolamine phospholipase D (NAPE-PLD) [16]. Additional NAEs, such as *N*-palmitoylethanolamine (PEA) and *N*-oleoylethanolamine (OEA), are also produced via NAPE-PLD [17]. However, the existence of enzymatic activity capable of converting NAPE to AEA in a calcium-independent manner in NAPE-PLD−/− mice [18] suggested the existence of additional, parallel biosynthetic pathways involving the recently identified αβ-hydrolase 4 (Abhd4) and the putative tyrosine phosphatase PTPN22 [19,20]. 2-AG is mostly synthesized from membrane phospholipids via a two-step process catalyzed by a phospholipase (PLCβ) and a diacylglycerol lipase (Di Marzo et al. 1998). Alternative pathways involve the action of a PLA1 and of lyso-PLC [21] or the hydrolysis of LPA by an LPA phosphatase [22]. The lifespan of EC and NAE is limited by their enzymatic degradation. The enzymes mainly responsible for their catabolism are fatty acid amide hydrolase (FAAH), *N*-acylethanolamine acid amide hydrolase (NAAA), and monoacylglycerol lipase (MAGL). FAAH is considered the chief AEA/OEA-degrading enzyme, MAGL is considered the chief 2-AG-degrading enzyme, and NAAA is considered the main PEA-degrading enzyme. Additional serine hydrolases also contribute to 2-AG hydrolysis: FAAH, serine hydrolase α-β-hydrolase domain 6 (ABHD6), and serine hydrolase α-β-hydrolase domain 12 (ABHD12) [23,24]. Moreover, under certain circumstances, both AEA and 2-AG can be also oxygenated by cyclooxygenase 2 (COX-2) and lipooxygenase 12 (LOX12) to generate prostaglandin-ethanolamide or prostaglandin-glycerol-esters and hydroxyl-eicosatetraenoyl-ethanolamide or hydroxy-eicosatetraenoic-acid-glyceryl ester, respectively [25,26,27].

Abnormal bone phenotypes are reported in mice lacking either CB1 or CB2 receptors varying with age, gender, or genetic background [28,29,30,31,32,33,34], suggesting active participation of the endocannabinoid system in bone cell differentiation and function. Indeed, synthetic cannabinoid AM251, a CB_1_ receptor antagonist/inverse agonist, or CB1 genetic deletion decreased osteoblast differentiation capacity from bone marrow-derived cells [35]. Different studies highlighted also the therapeutic value of CB_2_ receptor ligands in supporting bone tissue formation and mineralization or inhibiting osteoclast formation, suggesting its importance for bone cell function [32,33,36,37]. In the context of bone, 2-AG and AEA were reported to be produced in murine trabecular bone [30,38] and human osteoclasts [39]. Some authors reported that treatment of rat bone marrow stromal cells (BMCs) with 2-AG increased alkaline phosphatase (ALP), a marker of osteoblast differentiation [40]. On the contrary, other authors [30] reported that 2-AG has no effect on ALP production and cell number in a murine osteoblast cell line MC3T3-E1 [30]. Moreover, AEA and 2-AG, exogenously given, presented time-dependent effects on the differentiation of human osteoblasts and exhibited different roles during osteoblast maturation and matrix mineralization. In particular, AEA increased osteoblast differentiation marker alkaline phosphatase (ALP) in the early stage, and 2-AG increased osteoblast-specific marker osteocalcin in the early stage but decreased it in the late stage [41]. There are many conflicting data regarding the exact involvement of cannabinoid receptors in bone mass and bone remodeling process. Some studies highlighted the presence and importance of endocannabinoids (AEA and 2-AG) in bone tissue and during osteoclast differentiation from monocytes. However, comparatively little is known regarding the endogenous cannabinoid levels, including the expression of their metabolic machinery during osteoblast formation and maturation. In addition, there are no data yet regarding the modulation of ECs and related molecules within bone tissue between the early and late periods of skeletal development (specifically from infancy up to adulthood). Therefore, in the present study, we aimed at investigating changes in endocannabinoids and EC-related molecules in femurs of B6D2male mice at ages characterized by highly active bone growth. Considering the complexity of this biological sample due to the presence of bone marrow, we also characterized the modulation of this endogenous system particularly during all differentiation phases of the murine calvarial pre-osteoblast cell line MC3T3-E1.

## 2. Materials and Methods

### 2.1. Animals

Male mice on a mixed background (C57BL/6xDBA/2, hereafter referred to as B6D2) purchased from Charles River Laboratories were used for the study. Animals were housed 3–4 to cage under standard conditions with a 12:12-h light/dark cycle and had food and water ad libitum. Studies were carried out in accordance with the National Guidelines for Animal Use (authorization n.551/2015-PR released by the Italian Ministry of Health). All efforts were made to minimize the potential suffering and discomfort of animals, and care was taken according to the 3R rule (replacement, reduction, and refinement).

### 2.2. Cells Culture and Differentiation

The murine calvarial pre-osteoblast cell line MC3T3-E1 (subclone 4; ATCC, Manassas, VA, USA) was used in the study. Cells were cultured in growth medium (Minimum Essential Medium Eagle (alpha-MEM) without ascorbic acid (A10490-01, Gibco, Life Technologies, Carlsbad, CA, USA) supplemented with 10% FBS (Gibco, USA) and 1% penicillin–streptomycin (Life Technologies, Carlsbad, CA, USA). For osteoblast differentiation, cells were plated at an initial density of 3.5 × 10^4^ cells/well in 6-well plates. After reaching the confluence, cells were cultured for additional 21 days in growth medium supplemented with osteogenic factors 10 mM of β-glycerophosphate (Santa Cruz Biotechnology, Inc., Dallas, TX, USA) and 50 μg/mL of ascorbic acid (MilliporeSigma, Munich, Germany). The differentiation-inducing medium was replaced three times per week. Matrix mineralization was quantified by alizarin red staining. Monolayers of MC3T3-E1 cells in 6-well plates were fixed 1 h in 70% ethanol, rinsed, and stained for 30 min 40 mM alizarin red stain solution (ARS, MilliporeSigma, Munich, Germany) before being photographed.

### 2.3. LC-MS Quantification of Endocannabinoids and Related N-Acylethanolamines

Differently aged (1-, 3-, and 8-month-old) mice were sacrificed, and right femurs were dissected and stored until further procedure after immediate freezing under nitrogen. MC3T-E1 cell pellets and media were collected at different times (days 0, 7, 14, and 21) of the differentiation process and stored at −80 °C until lipid extraction. Samples (tissue and bone marrow) were homogenized in chloroform/methanol/Tris HCl 50 mM (2:1:1 by volume), containing 50 pmol of d^5^-2-arachidonoylglycerol (d^5^-2-AG) and 5 pmol of d^8^-anandamide (d^8^-AEA), 50 pmol of d^2^-oleoylethanolamide (d^2^-OEA), and 50 pmol of d^4^-palmitoylethanolamide (d^4^-PEA) as internal standards. Homogenates were centrifuged at 10,000× *g* rpm for 5 min and the aqueous phase plus debris was extracted four times with chloroform. The organic phase containing lipid extracts was dried, weighed, and later purified by using open-bed chromatography on silica gel with 99:1, 90:10, and 50:50 (*v*/*v*) chloroform/methanol. The 90:10 fraction was used for EC and *N*-acylethanolamine quantification by LC-APCI-MS (LCMS-2020, Shimadzu, Milan, Italy) as previously reported [42].

### 2.4. RNA Isolation and Quantitative Polymerase Chain Reaction (qPCR)

MC3T3-E1 cells (days 0, 7, 14, and 21) were collected in 1 mL of Trizol reagent (Invitrogen, Waltham, MA, USA) and frozen at −80 °C until further procedures. RNA isolation was performed according to the manufacturer’s protocol and to remove residual of contaminating genomic DNA, further digested by DNase I, Amplification Grade Thermo (Invitrogen, Waltham, MA, USA). The total RNA quantity was assessed using Eppendorf BioPhotometer (Eppendorf, Hamburg, Germany). Each sample was equalized to a concentration of 1 μg/μL and reverse transcribed to cDNA using SuperScript III First-Strand Synthesis System (Invitrogen, USA) according to the manufacturer’s protocol. The qPCR reactions were carried out using Green-2-Go qPCR Mastermix (Bio Basic Inc., Markham, ON, Canada). The reactions were run on a Real-Time PCR CFX384 Touch qPCR System (Bio-Rad Laboratories, Inc., Hercules, CA, USA). Samples were amplified simultaneously in triplicate and expression levels were assessed against housekeeping gene *B2m*. Cycle threshold values were calculated automatically by the CFX Manager software. mRNA abundance was calculated as 2^−(threshold cycle)^.

### 2.5. Statistical Analysis

All data are presented as mean ± SEM and analyzed by one-way analysis of variance (ANOVA) followed by Tukey’s post hoc test. For lipidomic analyses, two independent experiments were run for the in vitro data with *n* = 4 for each group at each time point. One experiment was performed for the in vivo data with *n* = 4 for each group at each time point. For qPCR analyses, sample size was *n* = 3 run in triplicate. Differences between sample groups were considered significant at a *p* value of <0.05. * denotes significant differences vs. day 0 or 1-month-old mice, **#** denotes significant differences vs. day 7, and $ denotes significant differences vs. day 14. Statistical analyses were performed with GraphPad Prism 8.3 (GraphPad Software, Inc., San Diego, CA, USA).

## 3. Results

### 3.1. mRNA Expression of Early and Late Markers during MC3T3-E1 Osteoblast Differentiation and Alizarin Red Matrix Mineralization Quantification

**Figure 1:**
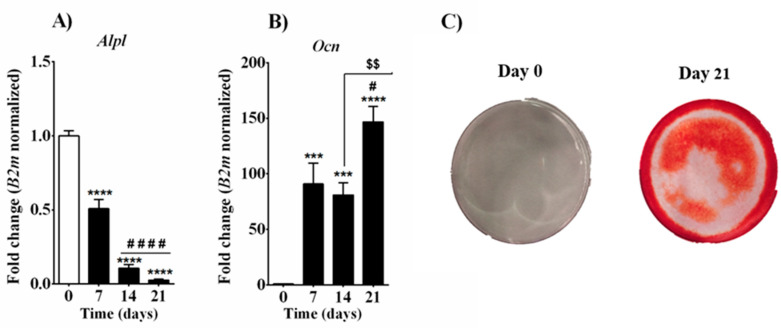
Differentiation process in MC3T3-E1 cells. Quantitative analysis of osteoblast differentiation markers Alp (**A**) and Ocn (**B**). Alizarin red staining for bone nodule formation (**C**). Samples were collected at different time points, before and after the induction of the differentiation process by supplementing medium with β-glycerophosphate and ascorbic acid. Data are presented as fold change (mean ± SEM) normalized to the expression of reference gene B2m and compared to basal group (day 0). Statistical analysis was performed using a one-way ANOVA followed by Tukey’s post hoc test, values with *p* < 0.05 were considered significant. * denotes significant differences vs. day 0; # denotes significant differences vs. day 7; $ denotes significant differences vs. day 14. Alizarin red staining was performed on days 0 and 21. Meaning of symbols: one denotes *p* < 0.05; two denotes *p* < 0.01; three denotes *p* < 0.001 and four denotes *p* < 0.0001.

Bone-forming osteoblasts are derived from mesenchymal stem cell precursors and undergo a defined maturational sequence starting from proliferating pre-osteoblasts to mature synthetically active and mineralized osteoblasts. Alkaline phosphatase (ALP encoded by *Alpl* gene) is an essential enzyme during the early stage of osteoblastic differentiation. Indeed, it is considered as a marker of early osteogenesis differentiation, while osteocalcin (*Ocn*), the most abundant protein in bone besides collagen, is observed during a later stage of osteoblast differentiation [43]. To confirm MC3T3-E1 cell differentiation into mature osteoblasts, analysis of *Alpl* and *Ocn* mRNA expression was performed. Pre-osteoblast MC3T3-E1 cells expressed the highest *Alpl* mRNA level during their proliferation and organic matrix production phases (day 0 and day 7, respectively), with a significant gradual lowering of its expression until their maturation phase at day 21 (Figure 1A). On the contrary, *Ocn* transcripts were undetectable before the induction of differentiation (day 0), but their levels were significantly raised during proliferation and matrix maturation phases, with a strong increase at day 21 that resulted in an approximately 150-fold change compared to basal level (day 0, Figure 1B). Osteoblast differentiation and bone nodule formation were confirmed by means of alizarin red staining. MC3T3-E1 cells showed their ability to produce a mineralized matrix after 21 days of growth in a differentiation-inducing medium (Figure 1C).

### 3.2. Quantification of Endocannabinoids and EC-Related Molecules in MC3T3-E1 Cells during Differentiation

**Figure 2:**
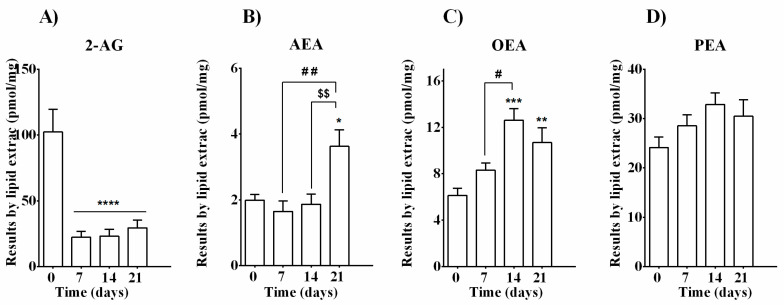
Evaluation of EC and NAE levels in MC3T3-E1 cells during 21 days of the differentiation process. LC-MS analysis was performed at different time points before (day 0) and after (days 7, 14, and 21) the induction of the differentiation process with cultured medium supplemented with β-glycerophosphate and ascorbic acid. Data are presented as mean ± SEM of pmol per mg of dry lipid extract for (**A**) anandamide (AEA), (**B**) 2-arachidonoylglycerol (2-AG), (**C**) oleoylethanolamide (OEA), and (**D**) palmitoylethanolamide (PEA). Statistical analysis was performed using a one-way ANOVA followed by Tukey’s post hoc test; *p* values < 0.05 were considered significant. * denotes significant differences vs. day 0; # denotes significant differences vs. day 7; $ denotes significant differences vs. day 14. Meaning of symbols: one denote *p* < 0.05; two denotes *p* < 0.01; three denotes *p* < 0.001 and four denotes *p* < 0.0001.

Murine calvarial pre-osteoblasts MC3T3-E1 cells were collected at day 0 or cultured in growth medium supplemented with osteogenic factors up to 21 days. To investigate the possible involvement of ECs and related molecules during the differentiation process of bone-forming cells, endogenous levels of 2-AG, AEA, OEA, and PEA were measured at different time points (days 0, 7, 14, and 21). All molecules were found detectable (Figure 2A–D), confirming the capability of these cells to produce them. Among all, 2-AG levels were the most abundant (102.4 ± 17.25 pmol/mg per lipid extract) before the differentiation process started (day 0). However, these levels drastically declined to approximately 25 pmol/mg per lipid extract throughout the differentiation (day 7) with persistent reduction until the maturation process was completed (day 14 and 21) (Figure 2A). Differently, detectable and unaltered levels of AEA (ranging from 1.65 to 1.98 pmol/mg per lipid extract) were found up to day 14, while a significant increase (3.62 ± 0.49 pmol/mg per lipid extract) was observed in mature osteoblast cells at day 21 (Figure 2B). Similarly, treatment with osteogenic factors increased basal OEA levels (6.138 ± 0.6115 pmol/mg per lipid extract, day 0) already after one week of exposure (8.314 ± 0.6022 pmol/mg per lipid extract, day 7), although not in a significant way. OEA levels were instead significantly raised at day 14 and day 21 (12.62 ± 0.9971 pmol/mg per lipid extract and 10.69 ± 1.275 pmol/mg per lipid extract, respectively), suggesting that OEA may be important for osteoblast maturation and matrix mineralization (Figure 2C). PEA levels ranged from 24.15 ± 2.126 pmol/mg (day 0) to 32.88 ± 2.341 pmol/mg (day 14) and 30.48 ± 3.378 pmol/mg (day 21), without any significant difference (Figure 2D).

### 3.3. Expression of 2-AG, AEA, OEA, and PEA Metabolic Enzymes during Osteoblast Differentiation in MC3T3-E1 Cells

**Figure 3:**
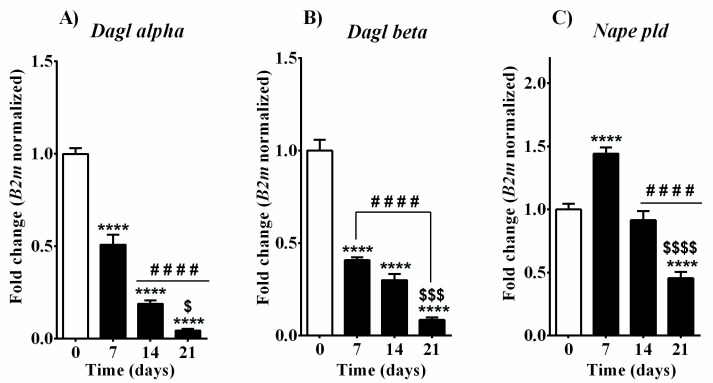
Analysis of mRNA expression of main EC and related NAE synthetic enzymes in MC3T3-E1 cells during the differentiation process. (**A**) *Dagl alpha*, (**B**) *Dagl beta*, (**C**) *Naple pld*. Samples were collected at different time points before (day 0) and after (days 7, 14, and 21) the induction of the differentiation process by cultured medium supplemented with β-glycerophosphate and ascorbic acid. Data are presented as fold change (mean ± SEM) normalized to the expression of reference gene 2-microglobulin (B2m) and compared to basal group (day 0). Statistical analysis was performed using a one-way ANOVA followed by Tukey’s post hoc test; *p* values < 0.05 were considered significant. * denotes significant differences vs. day 0; # denotes significant differences vs. day 7; $ denotes significant differences vs. day 14. Meaning of symbols: one denote *p* < 0.05; two denotes *p* < 0.01; three denotes *p* < 0.001 and four denotes *p* < 0.0001.

**Figure 4:**
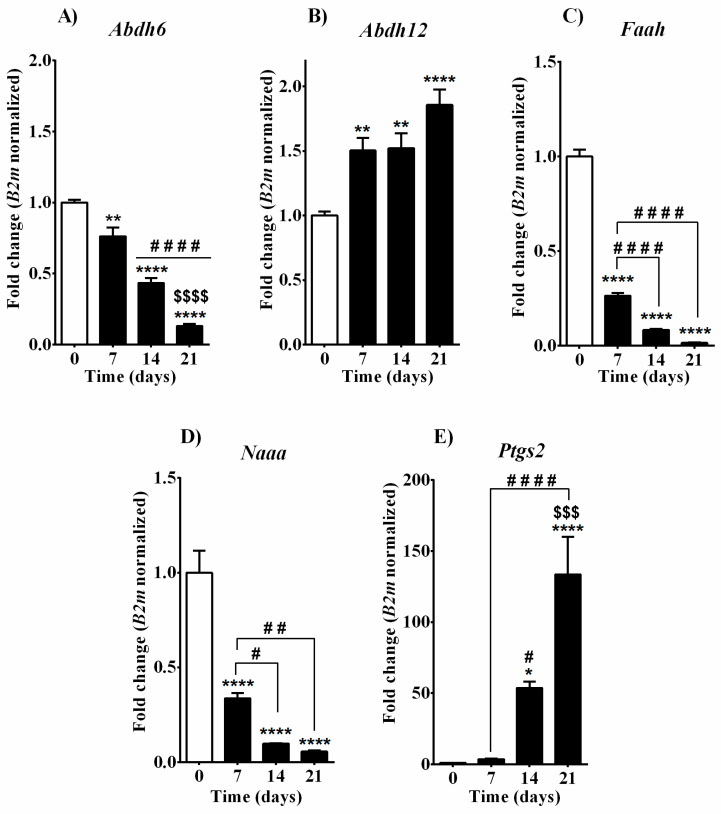
Analysis of mRNA expression of main EC and related NAE catabolic enzymes in MC3T3-E1 cells during the differentiation process. (**A**) *Abdh6*, (**B**) *Abdh12*, (**C**) *Faah,* (**D**) *Naaa*, (**E**) *Ptgs2.* Samples were collected at different time points before (day 0) and after (days 7, 14, and 21) the induction of the differentiation process by cultured medium supplemented with β-glycerophosphate and ascorbic acid. Data are presented as fold change (mean ± SEM) normalized to the expression of reference gene 2-microglobulin (B2m) and compared to the control group (day 0). Statistical analysis was performed using a one-way ANOVA followed by Tukey’s post hoc test; *p* values < 0.05 were considered significant. * denotes significant differences vs. day 0; # denotes significant differences vs. day 7; $ denotes significant differences vs. day 14. Meaning of symbols: one denote *p* < 0.05; two denotes *p* < 0.01; three denotes *p* < 0.001 and four denotes *p* < 0.0001.

In line with the decline observed in 2-AG levels, mRNA levels of the two main enzymes responsible for its synthesis—DAGL (encoded by Dagl alpha and Dagl beta genes respectively)—were gradually and significantly reduced over time during the differentiation process (Figure 3A,B). Concerning Nape pld, the key player in AEA, OEA, and PEA synthesis, the analysis of its transcript level showed a significant increase 7 days after the induction of the differentiation process with β-glycerophosphate and ascorbic acid and, later on, displayed a gradual decrease, reaching basal level at day 14, and substantial downregulation on day 21. Thus, contrarily from what was expected from the correspondent levels of its endogenous substrates, *Nape pld* enzyme expression increased during the intense osteoblast proliferation stage and decreased alongside the osteoblast maturation or mineralization (Figure 3C). When we investigated mRNA expression of the main enzymes responsible for AEA, OEA, and PEA hydrolysis (*Faah* and *Naaa*), we found a consistent decrease in MC3T3-E1 cells alongside all phases of osteoblast maturation (days 7, 14, and 21; Figure 4C,D). We also considered another enzyme (COX-2 encoded by the *Ptgs2* gene), which is responsible for the oxidative catabolism of both AEA and 2-AG. We found a strong upregulation in MC3T3-E1 cells as compared to basal levels with over 50- and 100-fold changes of *Ptgs2* transcripts after 14 and 21 days of supplementation with osteogenic factors, respectively (Figure 4E). The expression of 2-AG main degrading enzyme *Magl* was instead not detected in MC3T3-E1 cells in any of the differentiation phases (data not shown), suggesting that in MC3T3-E1 cells 2-AG catabolism and its possible role in osteoblast differentiation does not rely on MAGL enzyme. Therefore, we measured the expression of the two additional enzymes involved in 2-AG hydrolysis, *Abhd6* and *Abhd12* (Figure 4A,B). In particular, mRNA levels of *Abhd6* were gradually and enduringly decreased after day 7 (Figure 4A). *Abhd12* expression was continuously upregulated after induction of the differentiation process with higher expression at day 21 in mature osteoblast cells (Figure 4B).

### 3.4. Quantification of Endocannabinoids and EC-Related Molecules in Femurs of C57BL6 Male Mice

**Figure 5:**
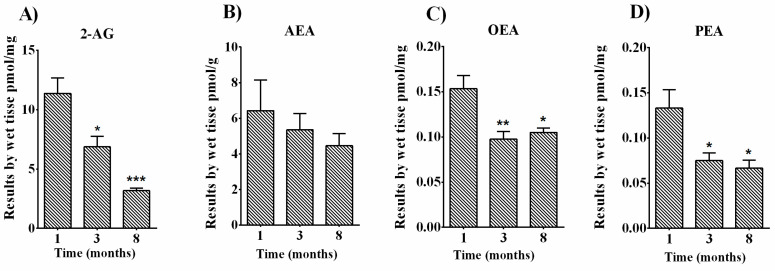
LC-MS analysis of ECs and NAEs in femurs of B6D2 male mice. Bone tissues for evaluation of (**A**) 2-arachidonoylglycerol (2-AG), (**B**) anandamide (AEA), (**C**) oleoylethanolamide (OEA), and (**D**) palmitoylethanolamide (PEA) levels were collected at 1, 3, and 8 months of age. Data are presented as mean ± SEM of pmol per mg (2-AG, OEA, and PEA) or pmol per g (AEA) of wet tissue weight. Statistical analysis was performed using one-way ANOVA followed by Tukey’s post hoc test; *p* values < 0.05 were considered significant. * *p* < 0.05, ** *p* < 0.01, *** *p* < 0.001 versus 1-month-old animals.

A targeted lipidomics approach was applied to quantify endocannabinoid and related *N*-acyl amide levels in femurs of B6D2 male mice at different months of age. LC-MS analyses confirmed the presence of the two major endocannabinoids AEA and 2-AG as well as the two major related-*N*-acylethanolamines OEA and PEA in mouse bone tissue. 2-AG displayed high levels (11.38 ± 1.296 pmol/mg of wet tissue) in 1-month-old tissues, which significantly declined over time (Figure 5A) (6.867 ± 0.8762 pmol/mg and 3.175 ± 0.2056 pmol/mg in 3- and 8-month-old mice, respectively). AEA displayed low levels (range of pmol/g of wet tissue) as compared with the other targets investigated (range of pmol/mg of wet tissue) in 1-month-old thighbones and showed a gradual decrement alongside aging, although not significant (Figure 5B). Similarly to 2-AG, OEA and PEA levels were also reduced in femurs of 3- and 8-month-old mice when compared to 1-month-old animals (Figure 5C,D). In particular, starting from 0.1533 ± 0.01453 pmol/mg in youngest mice, OEA levels declined to 0.0975 ± 0.008539 pmol/mg (at 3-months) and 0.1050 ± 0.005 pmol/mg (at 8-months, Figure 5C). PEA levels in 1-month-old mice were detected at 0.1333 ± 0.02 pmol/mg and, like OEA levels, were significantly reduced as mice aged, reaching 0.0750 ± 0.008 pmol/mg (3 months) and 0.6667 ± 0.008 pmol/mg (8 months, Figure 5D). Thus, 2-AG, OEA, and PEA levels were generally lowered within the bone tissue in older mice.

## 4. Discussion

Bone is a very complex and active tissue that undergoes renewal and repair throughout life and provides locomotion mechanical support and protection for vital organs. Osteoblasts undergo a defined maturational sequence, which starts from proliferating pre-osteoblasts and leads to mature, synthetically active, osteoblasts to finally become embedded within the bony matrix bone lining cells, osteocytes [43]. Osteoblasts express alkaline phosphatase, considered an essential enzyme in the early stage of osteoblastic differentiation, and osteocalcin, recognized as a marker for the middle or late stage of osteoblast differentiation [43]. Murine pre-osteoblast MC3T3-E1 cells are widely used in bone research. Despite variable osteogenic performance reported between subclones types, this cellular model has been cited over 5600 times since its introduction in 1981 [44,45,46,47]. Subclone 4, the same used in the present study, was reported to undergo temporal changes from proliferation to nodule formation and mineralization, expressing high levels of osteoblast marker mRNAs that positively affect in vitro mineralization as well as intramembranous osteogenesis in vivo [47,48]. Recently, a transcriptomic characterization of signaling pathways associated with osteoblastic differentiation of MC3T3-E1 cells was described [49]. Expression analysis of genes involved in multiple signaling pathways was correlated with the three stages of the differentiation process (growth arrest on days 2–5, differentiation on days 5–10, and osteoblast maturation on days 10–28) [49]. In the present study, when placed in culture medium supplemented with β-glycerophosphate and ascorbic acid for the following 21 days, MC3T3-E1 cells displayed differences over time in mRNA expression of well-known late and early differentiation markers *Alpl and Ocn* (Figure 1). Differentiating MC3T3-E1 cells expressed both genes, with higher *Alpl* transcript levels during the first phase of the differentiation process (day 0 and day 7), which gradually lowered until day 21 (Figure 1A). On the contrary, *Ocn* transcripts were low at the beginning of the differentiation process (day 0), but their levels significantly and gradually raised alongside the differentiation process up to day 21 (Figure 1B). In addition, alizarin red staining unveiled calcium deposits on osteoblast in MC3T3-E1 cells at day 21, confirming successful bone nodule formation (Figure 1C). As for *Alpl* expression, differently from our data, Luttrell and co-authors showed a gradual increase in mRNA levels between days 2 and 28 [49]. This discrepancy can be justified by the different experimental approaches used and the different time points analyzed. Evidence is reported showing that MC3T3-E1 cells exhibit increased ALP activity after reaching a confluent state, therefore supporting the idea that *Alpl* is also expressed in undifferentiated confluent cells [44,47]. A different study showed a variety of mRNA expression levels for ALP in undifferentiated and differentiated stages (day 12 of differentiation with osteogenic medium) among various MC3T3-E1 cell clones. Clone 4 in particular did not express mRNA for ALP at day 12 and showed high mRNA levels of OCN [48]. Sugawara et al. demonstrated that the activity of alkaline phosphatase is indispensable for the mineralization of MC3T3-E1 cells [50]. The bone nodule formation and mineralization observed in our cells by means of alizarin red staining demonstrate the accomplishment of osteoblast maturation most likely associated with ALP activity.

The osteoblast-specific process of bone formation is characterized by high energy demands due to the secretion of matrix proteins and mineralization vesicles. Independently from alkaline phosphatase, osteocalcin, and other well-known transcription factors, several endogenous molecules, including lipid mediators, have been reported to play a role in bone formation [43,50,51]. The principal fuel source for osteoblast differentiation is glucose; however, recent evidence indicates that bone-forming cells can utilize fatty acids as well [52,53,54]. After the identification of both CB1 and CB2 receptors in bone mass [28,29,30,31,32,33,34], a real interest in the role of the endocannabinoid system in skeleton and bone cell functions, including the differentiation process, has been born. *N*-acyl amides, fatty acid derivatives such as AEA and OEA, and oleoyl-serine have been found in the trabecular bone and reported as important regulators of the bone remodeling process and maintenance of skeletal homeostasis [55]. Other authors also reported detection of AEA and 2-AG in whole bone in mice, where levels of 2-AG were comparable to those found in the brain [28,30]. Conflicting data have been reported regarding the effects of exogenous 2-AG and/or AEA on alkaline phosphatase (ALP) and consequently on osteoblast differentiation in rat, mouse, and human [30,40,41]. Other authors focused more on the endogenous cannabinoid tone. Rossi et al. reported that human osteoblasts obtained from bone marrow of healthy donors express all the enzymatic components necessary for synthesis and degradation of AEA (NAPE-PLD and FAAH) and 2-AG (DAGL and MAGL) [56]. The expression of these enzyme patterns was reported also in MC3T3-E1 osteoblast-like cell line (subclone 4). In particular, higher expression of NAPE-PLD, FAAH, and CB2 mRNA was reported in mature osteoblasts (day 20) with respect to non-mature osteoblasts (day 10) [57]. Endocannabinoids are secreted on demand by osteoblasts, as occurs in other cellular systems. However, we expect that this “demand” changes over time during the osteoblast differentiation process depending on the possible role that each molecule can play in respect to the specific phase. In other words, endocannabinoids may exert a dynamic function throughout the differentiation steps. To the best of our knowledge, to date, there are no studies describing how the levels of endocannabinoids and especially of their related molecules are modulated in osteoblasts during different phases of differentiation. Therefore, in the present study, we aimed to quantify AEA, 2-AG, PEA, and OEA levels in MC3T3-E1 cells by measuring regular intervals of time (every 7 days) to acquire information on all the different stages of osteoblast development in vitro. Assuming a different on-demand production of these molecules over time, the transcriptomic expression of the enzymatic machinery necessary for their metabolism was also measured. Interestingly, 2-AG, AEA, OEA, and PEA levels in MC3T3-E1 pre-osteoblasts showed different modulation patterns during their differentiation into mature osteoblasts as measured before (day 0) and after (days 7, 14, and 21) media supplementation with β-glycerophosphate and ascorbic acid (Figure 2). Among all, 2-AG levels were highly abundant before the differentiation process started (day 0) and exhibited a drastic decline throughout the differentiation process until the completion of matrix mineralization (day 21). Inversely, detectable and unaltered levels of AEA were found up to day 14, while a significant increase was observed in mature osteoblast cells at day 21. We detected PEA levels making it the second most abundant molecule in differentiating MC3T3-E1 cells, although without significant changes between the different time points considered. Treatment with osteogenic factors increased basal OEA levels after one week of exposure, although not in a significant way. OEA levels instead were significantly raised at day 14 and day 21. The exact role of this increment remains to be determined, but it suggests that OEA can be important in osteoblast maturation and matrix mineralization. Alongside the endogenous levels detected via LC-MS, the transcript levels of *Nape pld* gene encoding a key producing enzyme not only for AEA but also for OEA and PEA were found in MC3T-E1 cells at all time points measured. *Nape pld* expression significantly increased in the first days of osteoblast development as measured on day 7 and decreased alongside osteoblast maturation/mineralization (day 14 and 21, Figure 3C). We found increased AEA and OEA levels at day 21, while *Nape pld* was significantly downregulated. The enzymatic regulation of AEA and OEA production is worthy of further investigation. A possible explanation for the discrepancy between AEA/OEA contents and their biosynthetic enzyme expression might be due to the contribution of alternative synthetic pathways such as the PLC pathway or the secreted PLA2 pathway [17,20,58,59]. Studies demonstrated that osteoblasts (rat calvarial cells cultured for 21 days) exhibit augmented PGE2 production during the mineralizing phase due to IL-1b-induced cPLA2, sPLA2, COX-2, and PGE synthase activities [60].

Assuming that alternative biosynthetic pathways are taking action, the high levels of AEA and OEA might be attributed to a reduced catabolic metabolic rate as we found lower levels of *Faah* transcripts in immature MC3T3-E1 cells at the early stage (day 7), which were consistently downregulated towards their maturation process (day 14 and 21, Figure 4C). Similarly, the expression of *Naaa* gene encoding for the enzyme that hydrolyzes PEA [61] showed a continuous downregulation in MC3T3-E1 cells alongside all phases of osteoblast development (Figure 4D). The downregulation of *Naaa* may partially be responsible for the unaltered PEA levels, which were kept around 29 pmol/mg at all measured time points in differentiating MC3T3-E1 cells. We also found a gradual time-dependent downregulation of the two genes *Dagl alpha* and *Dagl beta* encoding for biosynthetic enzymes DAGL alpha and beta, respectively (Figure 3A,B), which was consistent with the robust decline in 2-AG levels observed after the induction of the differentiation process. On the other hand, 2-AG levels are also regulated by monoacylglycerol lipase (MAGL) activity, which leads to its catabolism. In MC3T3-E1 cells, we were unable to detect *Magl* expression at any time points investigated. Two additional enzymes, the serine hydrolase α-β-hydrolase domain 6 and 12 (ABHD 6/12), were described to hydrolyze 2-AG [23,24], and we found them to be expressed by MC3T3-E1 cells. In particular, mRNA levels of *Abhd6* were decreased after day 7 (Figure 4A), while *Abhd12* expression was upregulated after the induction of the differentiation process (Figure 4B). We hypothesize that, in MC3T3-E1 cells, ABHD12 might act as an accessory enzyme that is recruited in the presence of particularly high amounts of 2-AG or when MAGL is unavailable. Endocannabinoids are also substrates for cyclooxygenase-2 (COX-2), an inducible enzyme that converts arachidonic acid to prostaglandins. The oxygenation of 2-AG and AEA via COX-2 generates prostaglandin glyceryl esters (PG-Gs) and prostaglandin ethanolamides (PG-EAs), respectively [62,63]. In our experimental settings, we found profound upregulation, over a 100-fold change in *Ptgs2* transcripts in mature osteoblasts (day 21) with respect to pre-osteoblast cells (day 0, Figure 4E). The increment of *Ptgs2* mRNA levels began as early as 14 days after differentiation process induction, suggesting that its expression may be relevant for osteoblast maturation and matrix production. In addition, the upregulation of COX-2 together with *Abdh12* starting at day 7 may contribute to 2-AG degradation, consistently with the decline in 2-AG levels observed alongside MC3T3-E1-derived osteoblast development. Recent studies highlighted the importance of COX-2 for bone formation and bone cell biology. Wasnik and co-workers showed that local COX-2 overexpression enhances MSC differentiation into osteoblast progenitors in niches near bone fracture sites, supporting bone fracture healing. In addition, prostanoids secreted by these COX-2-overexpressing cells elevated the expressions of osteocalcin in bone marrow-derived MSCs, which promoted osteoblast differentiation and suppressed chondrocyte differentiation in a way reverted by celecoxib, a COX-2-specific inhibitor [64]. In a different study, Chen et al. reported that prostaglandin E2 (PGE2) secreted by osteoblastic cells within the bone niche of active bone remodeling areas facilitates sensory nerve-stimulated bone formation, thus promoting bone regeneration [65]. All these findings suggest that COX-2 plays an important role in bone homeostasis with regard to the recruitment of bone-forming cells and their differentiation from precursors present within bone marrow and bone niches. In line with that, human mesenchymal stem cells treated with COX-2 inhibitors were reported to increase their ability to differentiate into adipocyte lineage rather than into osteoblast lineage, supporting the idea that COX-2 blockade prevents osteoblast differentiation and impairs the bone remodeling process [66].

Regarding bone marrow environment and bone, studies conducted by Idris et al. emphasize the importance of the endocannabinoid system in bone mass regulation and bone cell metabolism [35]. Authors showed that genetic and pharmacological modulation of CB1 receptors exerts bidirectional effects on bone mass at different stages in life (3, 6, and 12 months of age) by modulating osteoclast differentiation and by regulating differentiation of MSCs into osteoblasts or adipocytes [35]. In vivo studies performed on CB1−/− mice showed a reduction of bone formation when compared to wild-type mice and showed a gender-independent development of osteoporosis with aging [35]. MSCs derived from these CB1−/− mice showed a reduced ability in vitro to differentiate into osteoblasts and complete bone nodule mineralization but an increased capacity of differentiating into adipocytes [35]. CB2−/− mice showed accelerated age-related loss of trabecular bone and cortical expansion, though cortical thickness was reported to be unaltered [33]. This phenotype is also characterized by increased activity of trabecular osteoblasts (bone-forming cells), increased osteoclast (the bone-resorbing cell) number, and a markedly decreased number of diaphyseal osteoblast precursors. It is clear that both cannabinoid receptors have distinct roles in bone homeostasis and that their individual blockage may be harmful. ECs and NAEs act also on other molecular targets; therefore, cannabinoid receptors are not the only actors involved in cell differentiation. GPR18, GPR55, and PPARs are reported to be expressed in bone cells and involved in the regulation of bone mass homeostasis [67,68,69]. Another alternative target is the vanilloid receptor (TRPV1) [70,71]. TRPV1−/− mice show higher bone mass density, presenting osteoclast precursors that poorly respond to osteoclastogenic stimulus [72].

As a dynamic organ constituted by different cell types, all fundamental for maintaining bone homeostasis and in answering to mechanical stresses, bone undergoes a fine regulation by endogenous mediators like endocannabinoids and related molecules. The fact that cannabinoid receptors are co-expressed in mouse and human bone with vanilloid receptors suggests that endogenous ligands might act together to balance bone mineralization and resorption. In particular, molecules like AEA, being capable of activating both targets, can act via different actions on these receptors [73]. AEA and 2-AG production was reported in mouse femurs [28,30]. However, no studies are reported on the modulation of these endocannabinoids and related molecules, particularly PEA, in bone tissues from mice at different developmental stages (juvenile, young adult, and mature adult bone). Here we quantified AEA, 2-AG, OEA, and PEA levels in whole femurs from 1-, 3-, and 8-month-old B6D2 male mice. LC-MS analysis revealed that 2-AG contents (by mean of pmol/mg) were the most abundant in this tissue. However, except for AEA whose levels remained unaltered during all the different developmental ages analyzed, 2-AG, OEA, and PEA levels significantly declined in a time-dependent manner (Figure 5A–D). The underlying reasons for this time-dependent reduction require further investigations. Since this quantification was made on the whole femur (including bone marrow), which is mainly composed of osteocytes derived from osteoblasts and precursor cells that can differentiate in osteoblasts, we can only hypothesize that, in these femurs, osteoblast cells are involved in the modulation of 2-AG, OEA, and PEA. We speculate that 2-AG, OEA, and PEA are important for bone remodeling by affecting not only the recruitment of osteoblast precursor cells and their further differentiation but also the commitment of adipocytes or osteoclasts. Both AEA and 2-AG are produced by human osteoclasts [39]. Endogenous cannabinoid levels are reported to be modulated during the differentiation of osteoclasts from monocyte precursors with 2-AG decrement and AEA increment during the differentiation of mononuclear cells into multinucleated osteoclasts [74]. A recent study showed that OEA suppressed osteoclast cytoskeletal organization and bone resorption and induced apoptosis of mature osteoclasts [75]. Therefore, OEA secreted by osteoblasts may be a new interesting and potential target as a regulator of osteoclast activity. The hypothesis of an OEA-mediated regulation of bone cells is consistent with our results as we found decreased OEA tone in femurs of 3- and 8-month-old male mice (Figure 5C) with respect to 1-month-old mice. We can speculate about potential higher osteoclast activity due to decreased OEA levels in these femurs as old mice have been reported to exhibit excessive bone remodeling process together with bone loss [6,10]. However, all the above-mentioned hypotheses remain to be further investigated and were not under the scope of this work.

## 5. Conclusions

In the present study, we demonstrated the modulation of ECs and NAEs in pre-osteoblast MC3T3-E1 cells at various stages of their maturation process, and we showed a dynamic regulation during in vitro differentiation of their metabolic enzyme expression (Table 1).

We also reported 2-AG, OEA, and PEA decline in mouse femurs and bone marrow at different ages (young adult, and mature adult bone, Table 2). No other parameters were considered, and the role of other factors like receptors and/or enzymes remains to be established in this context. Nevertheless, several hypotheses were raised, setting the stage for further investigations on the local influence exerted by these molecules on bone turnover.

All these findings expand the existing knowledge regarding ECs and NAEs in the skeleton, underlying additional implications for physiological regulation of bone mass with an emphasis on bone-forming cell functions.

## Figures and Tables

**Table 1:** Summary of EC and NAE levels and gene expression alterations in MC3T3-E1 cells during the differentiation process.

		Days of Osteoblast Differentiation		
Investigated Group/Target		Day 7	Day 14	Day 21
EC and NAE levels	2-AG	↓↓↓↓	↓↓↓↓	↓↓↓↓
	AEA	-	-	↑
	OEA	-	↑↑↑	↑↑
	PEA	-	-	-
EC synthesis	*Dagl alpha*	↓↓↓↓	↓↓↓↓	↓↓↓↓
	*Dagl beta*	↓↓↓↓	↓↓↓↓	↓↓↓↓
	*Naple pld*	↑↑↑↑	-	↓↓↓↓
EC degradation	*Abdh 6*	↓↓	↓↓↓↓	↓↓↓↓
	*Abdh 12*	↑↑	↑↑	↑↑↑↑
	*Faah*	↓↓↓↓	↓↓↓↓	↓↓↓↓
	*Naaa*	↓↓↓↓	↓↓↓↓	↓↓↓↓
	*Ptgs2*	-	↑	↑↑↑↑

EC and NAE levels and relative mRNA levels of selected genes involved in their synthesis and degradation in MC3T3-E1 cells during the differentiation process. Lipid and gene expression levels were assessed by LC-MS analysis and qPCR, respectively. Samples were collected before (basal group, day 0) and 7, 14, and 21 days after induction of the differentiation process with cultured medium supplemented with β-glycerophosphate and ascorbic acid. Data were analyzed using a one-way ANOVA followed by Tukey’s post hoc test. ↑/↓ indicate significant differences vs. basal group (day 0). ↑ or ↓ for *p* < 0.05, ↑↑ or ↓↓ for *p* < 0.01, ↑↑↑ or ↓↓↓ for *p* < 0.001, ↑↑↑↑ or ↓↓↓↓ for *p* < 0.0001.

**Table 2:** Summary of EC and NAE alterations in femurs of B6D2 male mice.

		Age of B6D2 Male Mice (Months)	
Investigated Group/Target		3	8
EC and NAE levels	2-AG	↓	↓↓↓
	AEA	-	-
	OEA	↓↓	↓
	PEA	↓	↓

EC and NAE levels in femurs of B6D2 male mice collected at 1, 3, and 8 months of age. Statistical analysis was performed using one-way ANOVA followed by Tukey’s post hoc test. ↑/↓ indicate significant differences vs. 1-month-old animals. ↓ for *p* < 0.05, ↓↓ for *p* < 0.01, ↓↓↓ for *p* < 0.001.
